# Reconstituting the head and neck tumor microenvironment with air-liquid interface organoids

**DOI:** 10.3389/fonc.2025.1736505

**Published:** 2026-01-02

**Authors:** Luxi Zheng, Wei Tang, Shuqi Guo, Lin Chen, Shoupeng Wang, Feng Liu, Jian Meng

**Affiliations:** 1 The Xuzhou Clinical College of Xuzhou Medical University, Xuzhou Medical University, Xuzhou, China; 2 Department of Stomatology, Xuzhou Central Hospital, Xuzhou, Jiangsu, China; 3 School of Stomatology, Xuzhou Medical University, Xuzhou, China; 4 School of Stomatology, Shandong Second Medical University, Weifang, China

**Keywords:** air-liquid interface culture method, head and neck cancer, immune checkpoint inhibitors, immune microenvironment, organoids

## Abstract

**Introduction:**

A patient-derived head and neck cancer organoid (HNCO) model that can reconstruct the tumor-immune microenvironment (TME) was established using air-liquid interface (ALI) culture technology. The Tumor-Infiltrating Lymphocytes (TILs) and cancer-associated fibroblasts (CAFs) could be maintained in this model for a certain period of time. This model was confirmed to simulate PD-1/PD-L1 checkpoint blockade, providing a reliable *in vitro* model for the verification and clinical prediction of the therapeutic effects of relevant immunotherapy drugs for head and neck cancer (HNC).

**Methods:**

Fresh tumor tissue samples were obtained to establish an ALI head and neck cancer organoid (ALI-HNCO) model. The oncological characteristics of the organoids and their homology with parental tumors were verified using histomorphological analysis. T lymphocytes and fibroblasts in the organoids were detected using immunofluorescence staining. After treating with pembrolizumab (a PD-1 inhibitor), the secreted levels of the cytokine interferon-γ (IFN-γ) were measured using an enzyme-linked immunosorbent assay (ELISA), and changes in the ratio of CD8+/CD4+ distributed in the immune microenvironment of the organoid, as well as the expression of CD69+ immune cell subsets, were analyzed using flow cytometry. The FVS staining assay was used to verify the killing of tumor cells by cytotoxic T cells.

**Results:**

The comparison of immunofluorescence in organoids and parental tumor tissues showed that CD3+ lymphocytes and SMA+ cells were also present in the active organoid tissues. Approximately 17.86% (5/28) of the ALI-HNCO model could amplify specific reactive CD8+ T lymphocytes, generating tumor specificity and cytotoxicity.

**Discussion:**

An *in vitro* HNC immune microenvironment model was successfully constructed using the ALI method. This model maintained the proportions and structures of the components of the original tumor, such as tumor-infiltrating lymphocytes and cancer-associated fibroblasts, for a period of time *in vitro*, providing an experimental platform for exploring the complex crosstalk between HNC cells and multiple cell colonies. This study preliminarily validated the feasibility of using ALI organoid models to evaluate the efficacy of immunotherapy drugs in treating HNC, providing a reliable and stable preclinical model, and new ideas for drug screening platforms for personalized precision medicine in HNC.

## 1 Introduction

HNC is a highly prevalent malignancy worldwide. HNC refers to a group of malignant tumors located in the anatomical regions of the head and neck, including the oral cavity, larynx, pharynx and salivary glands. The main pathological type is squamous cell carcinoma (accounting for more than 90%), and also includes adenocarcinoma, mucoepidermoid carcinoma, undifferentiated carcinoma, ductal carcinoma, lymphoma, etc. (1–3) Currently, the primary treatment for HNC is surgery, supplemented by chemotherapy, radiotherapy, immunotherapy, and other combined treatment methods, which have greatly controlled the progression of the disease and improved the survival rate of patients (3–5). However, radiotherapy and chemotherapy have toxicity and many side effects, and the sensitivity of patients to treatment varies from person to person. Therefore, exploring new and efficient treatment methods with less toxic and fewer side effects, as well as individualized precision treatments, is of extremely significant importance for clinical therapy and neoadjuvant therapy before radical surgery.

Tumor tissue is a complex structure composed of multiple types of cells, which can continuously evolve and collectively form the tumor microenvironment (TME). The TME is composed of cellular components, such as tumor cells, cancer stem cells (CSCs), TILs, and CAFs, non-cellular components, as well as extracellular matrix (ECM) which are closely related to the occurrence, metastasis, and recurrence of malignant tumors (6, 7). The TME can control the proliferation and metastasis of tumor cells by transmitting signals through the autocrine-paracrine signaling pathway. By inducing immune tolerance and impairing the function of tumor-specific T cells, the TME promotes immune escape (8). A key mechanism for tumor immune escape is the upregulation of immune checkpoint molecules. Clinically, immunotherapy, exemplified by immune checkpoint inhibitors (ICIs) and adoptive cell therapy (ACT), has changed traditional paradigms of cancer treatment. Immune checkpoint blockade can activate anti-tumor immune responses. ICIs have brought significant clinical benefits to some tumor patients and shown extraordinary clinical application value. PD-1/PD-L1 blockade functions by releasing the inhibition of T-cells, thereby enhancing their activation and cytokine production against tumor cells (9–11). However, the number of patients suitable for this therapy is still very limited. Clinical studies on the first-line immunotherapy agent pembrolizumab have shown that the objective remission rate (ORR) of monotherapy in patients with R/M head and neck squamous cell carcinoma is about 17% (4, 12), and the ORR in advanced salivary gland cancer is about 4.6% (13). The main reason for these low rates is that patients may have congenital resistance to immunotherapy. Therefore, a new preclinical model that truly simulates the human tumor immune microenvironment must be established for the basic and clinical translational research of tumor immunity.

At present, 3D *in vitro* model technology is represented by organoid and tumor sphere models, and also includes air-liquid interface patient-derived organoids (ALI-PDO), microfluidic culture models, and 3D bioprinting models based on tissue engineering (14–18). Some previous *in vitro* 3D models lacked matrix components, including immune cells, which limited their application in studying the TME (19–21). However, recently, with the evolving progress and optimization of *in vitro* 3D culture technology and models, an increasing number of models have been established that can reshape the immune microenvironment *in vitro* and can be applied in fields such as patient efficacy evaluation and personalized treatment, drug screening (22), immunotherapy (23), and ACT studies (24). The ALI culture method is a special organoid culture model. Unlike the insufficiency of the co-culture model in evaluating the TME, the tumor organoids in this system retain the original tumor’s pathological features and genetic alterations while sustaining TILs and CAFs, thereby providing a superior model that closely mimics the *in vivo* tumor microenvironment (15). This method provides an overall strategy for *in vitro* immune TME modeling and can be used to explore the complex crosstalk among multiple different cell populations. Air-liquid interface patient-derived tumor organoids (ALI-PDTO) simulate the response process in tumor immunotherapy, which is of great significance for predicting the efficacy of and sensitization response to ICB therapy. The ALI culture of organoids has been reported globally in fields such as non-small cell lung cancer, adenocarcinoma, epithelioid sarcoma, and clear cell renal cell carcinoma (15, 25, 26). However, no studies have specifically focused on head and neck malignant tumors.

To reconstitute the tumor immune microenvironment of head and neck cancer *in vitro*, we established patient-derived head and neck cancer organoids using the ALI culture method. This model preserves tumor heterogeneity and simultaneously reconstructs key components of the TIME, allowing long-term maintenance of patient-derived immune cells and cancer-associated fibroblasts. Our study further demonstrated that ALI PDTOs can functionally simulate PD-1/PD-L1 checkpoint blockade. Treatment with the anti-PD-1 activated and proliferated tumor-infiltrating lymphocytes (TILs) in the ALI PDTOs, triggering a cytotoxic response. This model provides a valuable platform for deepening the understanding of tumor–immune microenvironment interactions, contributes significantly to tumor immunotherapy research, and holds potential for promoting the clinical translation of personalized immunotherapies.

## 2 Materials and methods

### 2.1 Sample source

All patients with HNC were recruited from Xuzhou Central Hospital. The eligibility criteria were as follows: patients with primary head and neck cancer who were scheduled for biopsy or lesion resection. We excluded those who had received any preoperative neoadjuvant therapy, including radiotherapy, chemotherapy, targeted therapy, immunotherapy, or other molecular treatments. The pathological types of all HNC samples were identified by the Pathology Department of Xuzhou Central Hospital, and the samples were then processed and tested. This study was approved by the Ethical Review Committee for Biomedical Research of Xuzhou Central Hospital. (Approval Number: XZXY-LK-20250115-0011) Before collecting specimens, the patients and their families provided signed informed consent.

### 2.2 Materials

The reagents and materials used in this study are listed in **Table 1**. All compounds were obtained commercially according to the specifications detailed in the table.

**Table 1:** Key resources table.

Reagent	Identifier	Source
Cellmatrix type I-A	CAT#: 637-00653	FUJIFILM Wako Pure Chemical Corporation
Recombinant human noggin protein	CAT#: HY-P7051A	MCE Corporation
Recombinant human R spodin-1 protein	CAT#: HY-P72784	MCE Corporation
Recombinant human Wnt-3A protein	CAT#: HY-P70453B	MCE Corporation
A83-01	CAT#: HY-10432	MCE Corporation
N-acetylcysteine (NAC)	CAT#: HY-B025	MCE Corporation
Human gastrin I	CAT#: HY-P097	MCE Corporation
Advance Dulbecco’s modified Eagle medium (DMEM)/F12 basic culture medium	CAT#: 12634-010	Thermo Fisher Scientific Inc
Ham's F-12 nutrient mixture powder	CAT#: 21700075	Thermo Fisher Scientific Inc
HEPES buffer	CAT#:15630-080	Thermo Fisher Scientific Inc
GlutaMAX	CAT#: 35050061	Thermo Fisher Scientific Inc
B27 supplement	CAT#: 17504-044	Thermo Fisher Scientific Inc
SB-202190	CAT#: 559388	Sigma-Aldrich
Nicotinamide	CAT#: 72340	Sigma-Aldrich
Human epidermal growth factor (EGF)	CAT#: HY-P7109	PeproTech Inc
Recombinant human interleukin (IL-2)	CAT#: 200-02	PeproTech Inc
Penicillin-streptomycin (Pen-Strep) glutamine	CAT#: 10378016	Invitrogen Corporation
Fixable Viability Stain 780	CAT#: 565388	BD Bioscience
Anti-CD45-percp-cy5.5	CAT#: 564106	BD Bioscience
Anti-CD3-PE-Cy7	CAT#: 560310	BD Bioscience
Anti-CD4-PE	CAT#: 561843	BD Bioscience
Anti-CD8-APC	CAT#: 561952	BD Bioscience
Anti-CD69-APC-R700	CAT#: 565155	BD Bioscience

### 2.3 Establishment of the HNCO model using the ALI method

**Figure 1:**
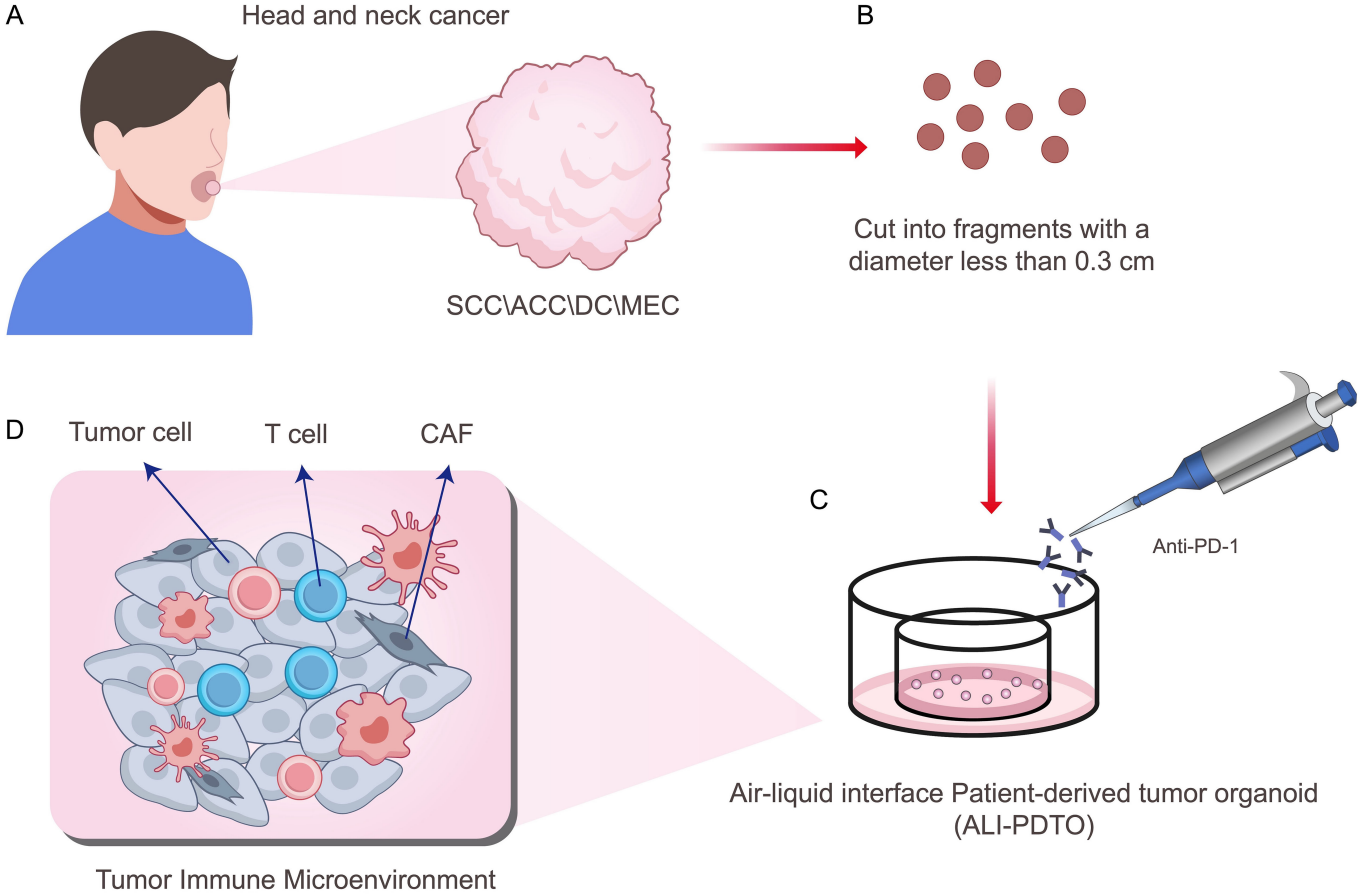
**(A)** Fresh human head and neck cancer tissues were surgically obtained. **(B)** Tumor tissues were physically minced using a non-enzymatic method. **(C)** Head and neck cancer organoids were cultured in an air-liquid interface dual-dish system. **(D)** The resulting model retained not only tumor cells but also lymphocytes and fibroblasts from the original tumor microenvironment.

Fresh HNC tissues were obtained through biopsy or surgical resection, stored in 20% fetal bovine serum (FBS)-DMEM, and transported on ice to our laboratory for ALI organoid culture within 2 h. The tumor tissue was cut into < 0.3 cm diameter fragments, mixed with recombinant collagen solution without enzymatic digestion, and evenly spread over pre-cured collagen gel in the internal Transwell insert with a permeable membrane at the bottom. The insert was placed in the cell culture dish and culture medium was added between the culture dish and the insert to form a dual-disc ALI culture system (**Figure 1**). In this way, the upper part of the tumor tissue and collagen mixture was directly exposed to air, and the culture medium penetrated the bottom of the collagen through micropores, forming an ALI culture model. ALI-HNCO collagen gel was mixed on ice with Cellmatrix I A solution, 10× Ham’s F-12 concentrated sterile medium, and sterile recombination buffer solution at a ratio of 8:1:1, containing final concentrations of 200 mmol/L HEPES, 0.05 mol/L NaOH, and 2.2 g NaHCO_3_ per 0.1 L (15).

The culture medium contained 1× Glutamax additive, 10 mmol/L HEPES, 1 mmol/L N-acetylcysteine, 10 mmol/L nicotinamide, 1× Pen-Strep glutamine, 1× B27, 10 μmol/L SB202190 (a p38MAPK inhibitor), 0.5 μmol/L A8301, 0.05–0.25 μg/mL R-spondin1 recombinant protein, 0.05–0.2 μg/mL recombinant human noggin protein, 50 ng/mL EGF, 10 nmol/L gastrin, 0.05–0.2 μg/mL human Wnt-3A, and 500 IU/mL IL-2 in Advanced DMEM/F12 basic culture medium.

### 2.4 Histological analysis

The mature ALI-HNCO was collected and fixed with 4% paraformaldehyde at 4°C for 30 min. The HNCO precipitate was blown to even distribution, followed by pre-embedding with liquid agarose, cooling to a gel-like state, and then subjected to gradient dehydration, transparency, paraffin-embedding, sectioning, and hematoxylin and eosin (H&E) staining and immunohistochemical (IHC) staining. After sealing, it was analyzed and compared with the corresponding original tumor tissue sections.

### 2.5 Immunofluorescence analysis

After the paraffin sections prepared in Section 2.4 were deparaffinized, antigen-retrieved, and subjected to three rounds of antibody staining. Each round included serum blocking, primary antibody incubation at 4°C overnight, HRP-labeled secondary antibody, and TSA-based fluorescent tyramide covalent labeling. The primary antibodies included CK5 (Servicebio, GB111246; 1:10000) for SCC, CK7 (Servicebio, GB12225; 1:2000) for MEC and DC, CD3 (Servicebio, GB12014; 1:2,000), and α-SMA (Servicebio, GB15044; 1:10000). The nuclei were counterstained with DAPI, and the sections were treated to quench autofluorescence, followed by sealing. Finally, the sections were sealed, and images were collected. This TSA-based method enables triple-labeling for protein co-localization analysis.

### 2.6 Addition of the immune checkpoint inhibitor pembrolizumab to activate T cells

PD-1 is a member of the B7/CD28 costimulatory receptor family and can be expressed on the surface of activated CD8+ T cells and B cells. It primarily regulates CD8+ T cell activity through binding to its ligands, PD-L1 and PD-L2, transmembrane proteins on tumor cells. Pembrolizumab has a high affinity for PD-1 on the surface of T cells and acts by blocking PD-1/PD-L1 cell channels, thereby facilitating cancer cell killing by the immune system. Since pembrolizumab is currently the first-line immunotherapy for HNC, the agent was chosen to explore the feasibility of using ALI-PDO models to evaluate the efficacy of immunotherapeutic drugs. IgG 4 (10 μg/mL) was the control group, and pembrolizumab (10 μg/mL) was the experimental group. We administered two consecutive doses of medication (changed every 3 days) to the cultured ALI organoids and cultured them for 7 days.

### 2.7 Secreted cytokine analysis by ELISA

The release of IFN-γ during T-lymphocyte activation was measured using the Human IFN-γ ELISA Kit. Cells treated with pembrolizumab and ICI were designated the experimental group, and IgG4 was designated the control group. The concentration of IFN-γ in culture medium was determined after 7 days. The average IFN-γ concentration was calculated by comparing the OD450 value of the sample measured on the microplate reader to the standard curve.

### 2.8 The activation of T cells and the killing of tumors detected by flow cytometry

The inner matrix gel was collected in a 15 mL centrifuge tube using precision tweezers, digested with 300 U/mL collagenase IV at 37°C for 30 min, followed by washing. The cell clusters were resuspended in 2 mL of Liberase TL (25 U/mL) and digested at 37°C for 15 min to prepare single cells. The single cells were washed once with MRS (5 mM EDTA/PBS) and FACS solution (PBS containing 2% FBS), respectively. Then, the cells were filtered, and the cell pellet obtained by centrifugation was resuspended in an appropriate amount of FACS solution. The sample was prepared by adding the following antibodies: Fixable Viability Stain 780, anti-CD45-percp-cy5.5, CD3-PE-Cy7, anti-CD4-PE, anti-CD8-APC, and anti-CD69-APC-R700. After staining, it was incubated on ice in the dark for 30 to 45 min. The sample was washed with 1 mL of FACS solution and resuspended in an appropriate amount of FACS solution or organoid growth medium (usually 100–500 μL). A total of 100,000 cells were collected using the LSRF Fortessa and analyzed using FlowJo software (version 10.8.1, Treestar).

### 2.9 Data analysis

Statistical analyses were carried out with GraphPad Prism, employing an independent samples t-test for two-group comparisons and one-way ANOVA for multi-group comparisons. Results are presented as follows: ns (not significant) for P > 0.05, *P < 0.05, **P < 0.01, ***P < 0.001, and ****P < 0.0001.

## 3 Results

### 3.1 Establishment and morphological validation of the immune microenvironment model for HNC

**Figure 2:**
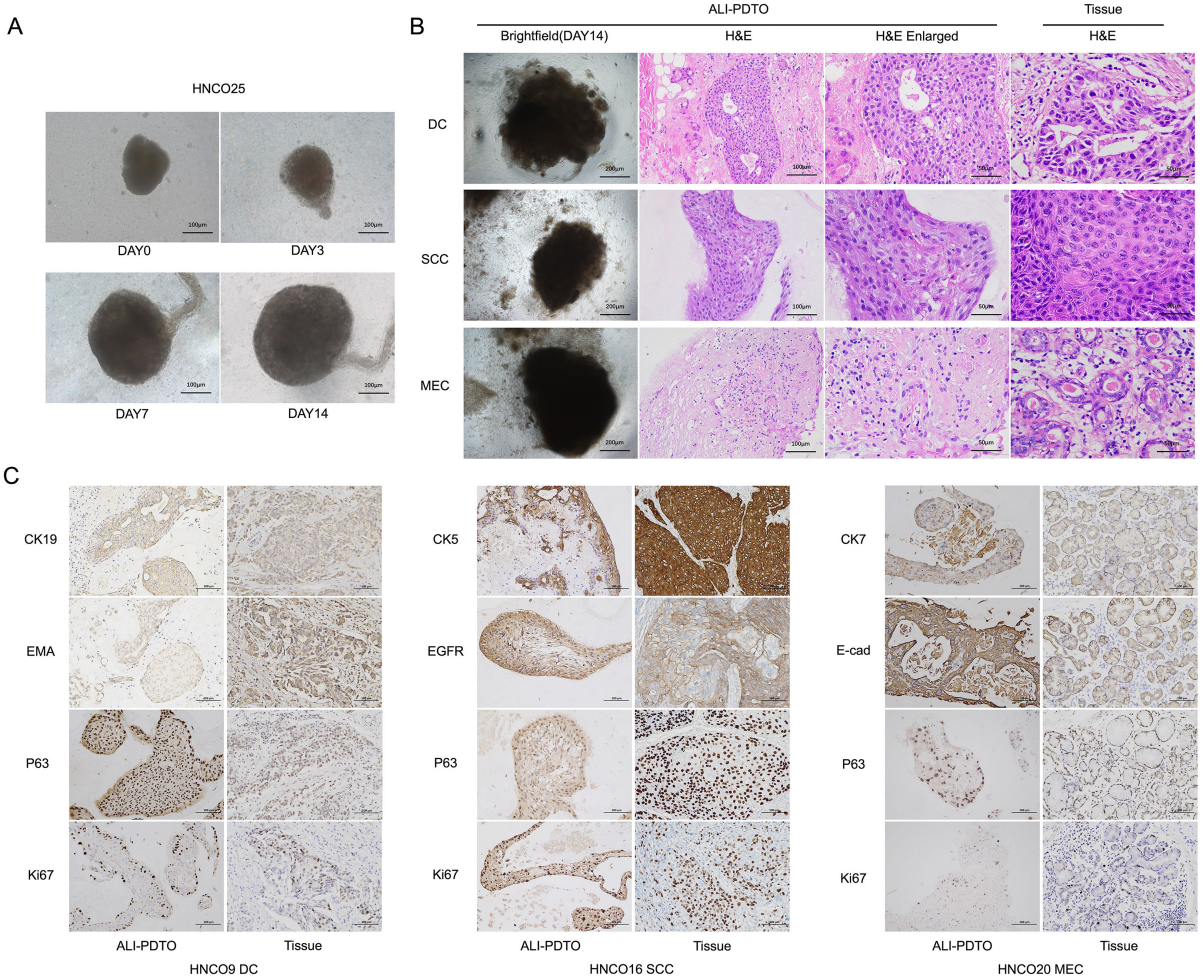
**(A)** Bright-field images of organoids taken under an inverted microscope on day 0, 3, 7, and 14. **(B)** Comparison of HNCO16 SCC/HNCO9 DC/HNCO23 MEC organoids on day 14, including inverted microscope images (200 μm), H&E staining images (100 μm, 50μm), and H&E staining images of their corresponding tumor tissues (50μm). **(C)** Immunohistochemical analysis images (100 μm) of HNCO16 SCC/HNCO9 DC/HNCO23 MEC organoids and their corresponding tumor tissues.

We established 28 HNCO models (28/35, with a success rate of 80%) using patient-derived HNC resection specimens. **Table 2** summarizes the detailed clinical and pathological data of the patients corresponding to HNCO. We successfully established an immune microenvironment model for HNC (**Figure 1**). Among the 35 cultured cases, 28 grew successfully, for an overall success rate of 80%. Within about 2 weeks of culture, the growth of organoids was recorded by bright-field photography using an inverted microscope (**Figure 2A**).

**Table 2:** Patient clinicopathological data of the 35 HNCO models.

ID	Gender	Age	Primary site	Histopathological subtype	Clinical TNM stage			Response to pembrolizumab
					T	N	M	
HNCO1	Male	72	Tongue	OSCC	3	0	0	Resistant
HNCO2	Female	68	Tongue	OSCC	4	0	0	Resistant
HNCO3	Male	81	Cheek mucosa	OSCC	2	0	0	Resistant
HNCO4	Male	65	Sublingual gland	MEC	3	0	0	Resistant
HNCO5	Male	77	Cheek mucosa	OSCC	3	2	0	Responsive
HNCO6	Male	56	Tongue	OSCC	3	0	0	Resistant
HNCO7	Female	63	Gingiva	OSCC	2	0	0	Resistant
HNCO8	Male	85	Tongue	OSCC	2	0	0	Resistant
HNCO9	Male	71	Parotid gland	DC	3	1	0	Responsive
HNCO10	Male	79	Gingiva	OSCC	2	0	0	Resistant
HNCO11	Female	66	Maxilla	OSCC	2	0	0	Resistant
HNCO12	Female	74	Tongue	OSCC	4	1	0	Resistant
HNCO13	Male	62	Mouth floor	OSCC	3	0	0	Resistant
HNCO14	Male	88	Cheek mucosa	OSCC	2	2b	0	Resistant
HNCO15	Female	59	Mouth floor	OSCC	2	0	0	Resistant
HNCO16	Male	83	Tongue	OSCC	3	0	0	Resistant
HNCO17	Female	75	Gingiva	OSCC	4b	0	0	Responsive
HNCO18	Male	64	Sublingual gland	DC	3	1	0	Resistant
HNCO19	Female	78	Tongue	OSCC	4a	0	0	Resistant
HNCO20	Male	53	Sublingual gland	MEC	2	0	0	Resistant
HNCO21	Male	61	Tongue	OSCC	3	1	0	Responsive
HNCO22	Male	73	Gingiva	OSCC	4a	1	1	Resistant
HNCO23	Female	67	Tongue	OSCC	4	0	0	Responsive
HNCO24	Male	82	Tongue	OSCC	2	1	0	Resistant
HNCO25	Male	70	Mouth floor	OSCC	2	0	0	Resistant
HNCO26	Male	76	Mandible	OSCC	2	1	0	Resistant
HNCO27	Female	84	Parotid gland	ACC	3	0	0	Resistant
HNCO28	Male	60	Tongue	OSCC	2	0	0	Resistant
N1	Female	89	Gingiva	OSCC	3	0	0	–
N2	Male	63	Gingiva	OSCC	3	0	0	–
N3	Female	71	Tongue	OSCC	2	0	0	–
N4	Male	75	Tongue	OSCC	2	2b	0	–
N5	Male	68	Gingiva	OSCC	3	0	0	–
N6	Male	80	Cheek mucosa	OSCC	4b	0	0	–
N7	Male	66	Tongue	OSCC	3	0	0	–

H&E staining and IHC assays were employed to analyze and compare the morphological and histological characteristics of HNCO and the corresponding tumor specimens. The nuclear atypia exhibited by HNCO was highly similar to that of homologous parent tumor tissues. CK5, EGFR, P63, and Ki67 staining of the SCC organoids was positive, which was consistent with those of the parents. CK9, EMA, P63, and Ki67 staining of the DC organoids were all positive, which was consistent with those of the parents. Among them, the staining results of CK7, E-cad, and P63 in one MEC organoid HNCO23 were consistent with those of its parental tissue/cells and showed positive signals, while Ki67 was consistent with the parents and showed negative results. That is, the results of typical IHC organoid markers were consistent with those of the parents, which can prove their homology at the histopathological level. The results also demonstrated the successful construction of HNCOs (**Figures 2B, C**).

### 3.2 The immune microenvironment model for HNC preserves the components and structure of the TME

**Figure 3:**
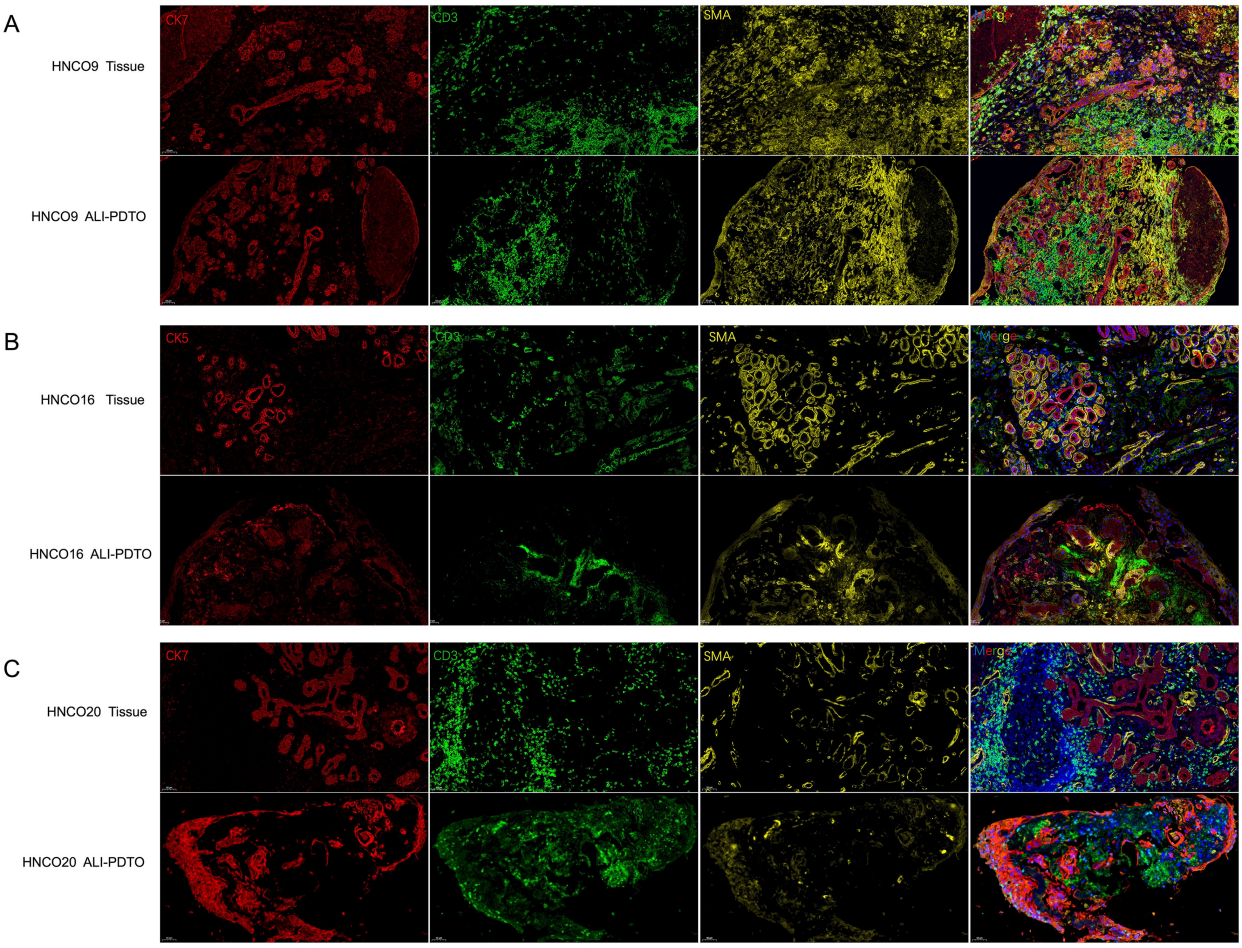
ALI-PDTOs were cultured until day 14 and subjected to immunofluorescence staining to verify their ability to retain the tumor microenvironment of the original tumor. **(A)** HNCO9 DC tumor tissue and its corresponding organoids: CK7 (red), CD3 (green), SMA (yellow), and nuclear DAPI staining (blue); **(B)** HNCO16 SCC tumor tissue and its corresponding organoids: CK5 (red), CD3 (green), SMA (yellow), and nuclear DAPI staining (blue); **(C)** HNCO23 MEC tumor tissue and its corresponding organoids: CK7 (red), CD3 (green), SMA (yellow), and nuclear DAPI staining (blue).

Immune cells, fibroblasts, and other non-tumor cells in the TME have a significant impact on tumor occurrence and development. Unlike the previous stromal cell scaffold method, the ALI-HNCO culture can maintain other components of the TME for a period of time. For this purpose, we performed immunofluorescence staining of parental tumor tissues and ALI-HNCOs cultured for 14 days to analyze the T-cell immune marker CD3 and the fibroblast immune marker SMA. The results showed that CD3+ cells and α-SMA+ cells could be preserved in ALI-HNCOs, confirming that the ALI-HNCO model can preserve T cells and fibroblasts in the TME (**Figures 3A–C**).

### 3.3 The HNC immune microenvironment model replicates immune checkpoint responses in the microenvironment

**Figure 4:**
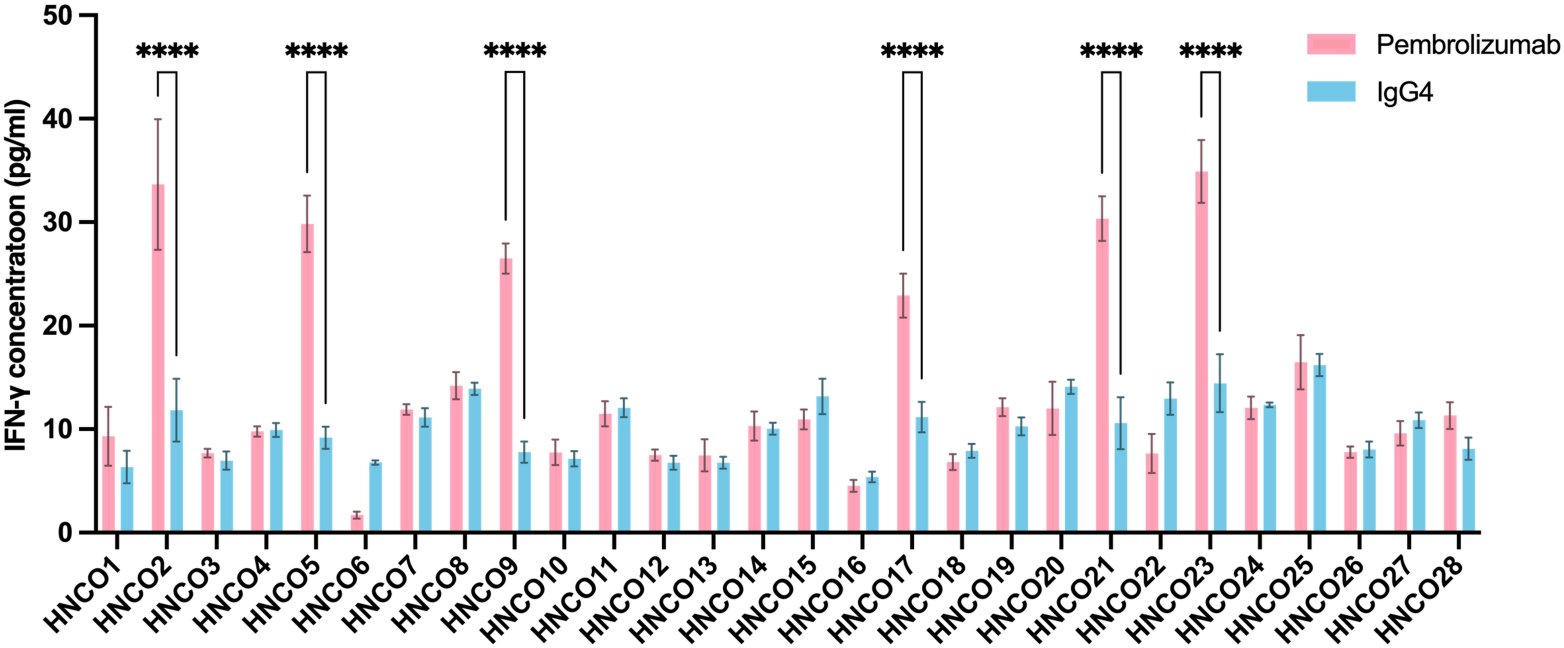
ALI-PDTOs recapitulated the response to immune checkpoint blockade therapy. After a 7-day treatment with Pembrolizumab in the experimental group and IgG4 in the control group, the concentration of IFN-γ in the culture medium was measured by ELISA. The results showed that the IFN-γ concentration in the experimental groups of HNCO2, HNCO5, HNCO9, HNCO17, HNCO21, and HNCO23 was significantly higher than that in the control groups, ****P<0.0001.

CTLs release effector cytokines, such as IFN-γ, which participate in the initiation and differentiation of CTLs and directly kill tumor cells (27). During tumor progression, T cells enter a state of exhaustion. The characteristics of exhausted T cells include the continuous high expression of multiple inhibitory receptors (e.g., PD-1) and the inability to secrete IFN-γ (28). Pembrolizumab effectively blocks the “brakes,” such as immune checkpoints (PD-1/PD-L1), enabling T cells to regain the ability to secrete IFN-γ. After 14 days of culture, the experimental group was treated with pembrolizumab, and the control group was treated with IgG4. Pembrolizumab mainly activated cytotoxic T cells (CD8+ T cells), while IFN-γ was mainly secreted by activated CD8^+^ CTLs. Therefore, we validated T-cell activation by measuring IFN-γ concentrations in the culture medium using ELISA. The ELISA results showed that IFN-γ concentrations in the six ALI-PDTO experimental groups increased significantly, revealing a remarkable difference compared with the control groups (**Figure 4**).

**Figure 5:**
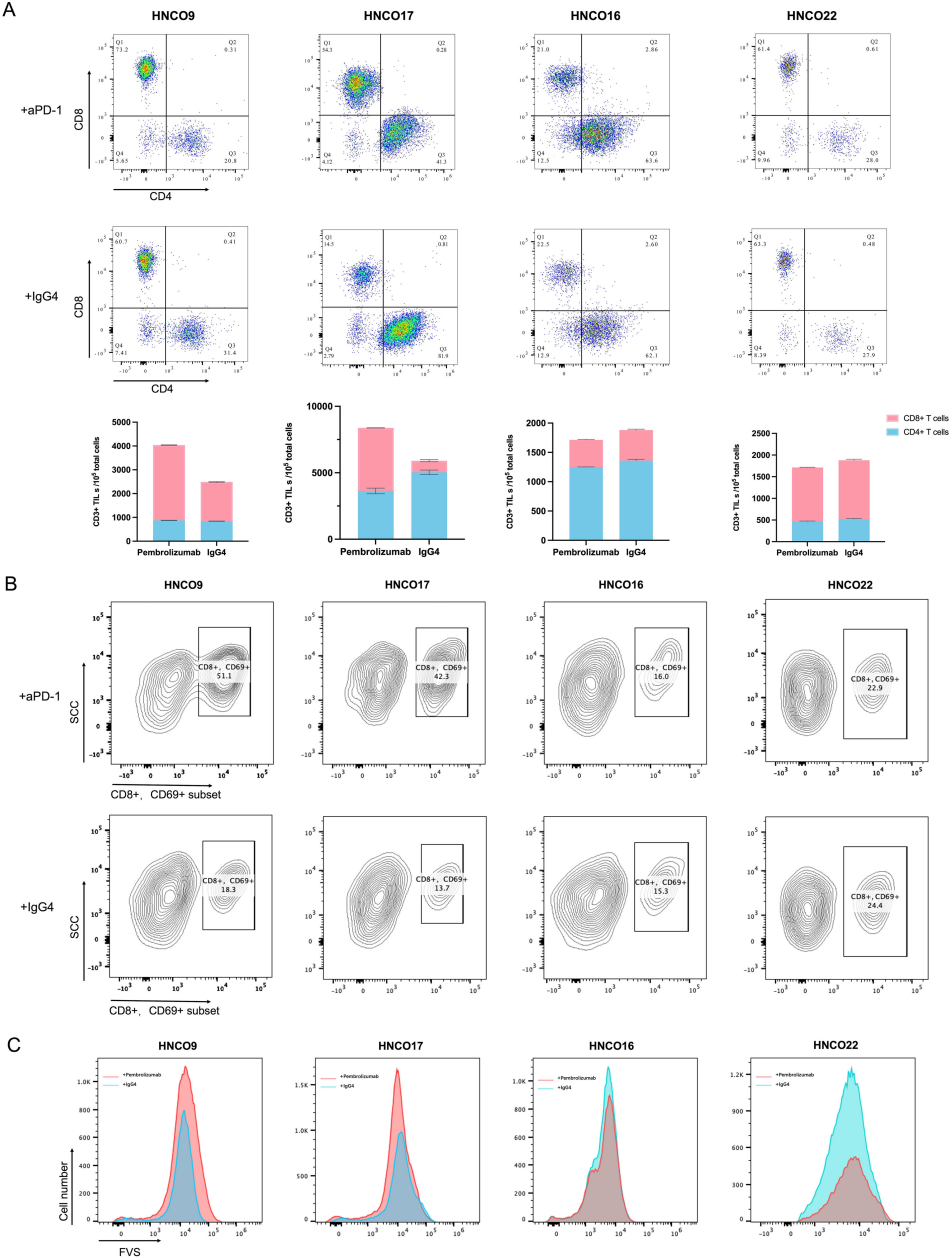
ALI-PDTOs recapitulated the response to immune checkpoint blockade therapy. After a 7-day treatment with Pembrolizumab in the experimental group and IgG4 in the control group, flow cytometry was performed to measure: **(A)** The ratio of tumor-infiltrating CD8+ T cell subsets to CD4+ T cell subsets in the experimental versus control groups; **(B)** The percentage of CD8+, CD69+ cell subsets within T cells in the experimental versus control groups; **(C)** A comparative histogram of tumor cells stained with FVS in the experimental versus control groups.

Pembrolizumab can rescue exhausted T cells. Due to the release of inhibition, antigen-specific CD8+ T cells undergo clonal proliferation, resulting in a significant increase in cell numbers. In contrast, the total number of CD4+ T cells remained relatively stable. Thus, the ratio of CD8+ to CD4+ naturally increased. An increase in the proportion of CD8+/CD4+ T cells was observed in five ALI-PDTO groups treated with pembrolizumab compared with the control group treated with IgG4, indicating that ICB rescued exhausted CD8+ T cells and reversed the immune depletion state of the TME. The IFN-γ concentrations in these five ALI-PDTO experimental groups increased significantly. However, in the remaining 23 groups, no significant change in the CD8+/CD4+ T-cell ratio was seen between the pembrolizumab-treated group and the IgG4-treated group, indicating the failure of CD8+ T-cell expansion after anti-PD-1 treatment (**Figure 5A**).

CD69 is an early marker of T-cell activation. Therefore, the expression of CD69 is a key signal for the successful activation of T cells and their transition from a quiescent state to a functional state (29, 30). Analysis revealed that in the five ALI-PDTO groups with elevated CD8+ T cells, the proportion of cytotoxic CD8+ and CD69+ cell populations among all T cells was significantly increased in the pembrolizumab-treated group compared with the IgG4-treated control group. In other organoid groups, no significant difference was seen in the proportion of CD69+ cells between the experimental and control groups (**Figure 5B**).

FVS staining of the cells after organoid digestion indicated that the organoid group, with a significantly increased proportion of cytotoxic CD69+ T cells, showed increased numbers of dead tumor cells, demonstrating anti-PD-1-dependent tumor killing activity (**Figure 5C**). This indicated that T cells were activated to exert a killing effect on tumor cells.

In conclusion, our results demonstrated the heterogeneity of responses to ICB. Among the 28 groups of organoids, five responded to pembrolizumab, while the remaining 23 were resistant to it. The OOR to the immunotherapy drugs was 17.86% (5/28), which was consistent with existing studies on OORs in HNCs, including squamous cell carcinoma and salivary gland cancer.

## 4 Discussion

We successfully established 28 ALI-HNCO models, which preserved the pathological features and genetic alterations of the original tumor. The ALI method has a higher utilization rate of tumor tissues than the traditional matrix gel-embedded culture method and does not require collagenase or trypsin to digest the tissue into individual cells, like traditional methods (31). The non-enzymatic digestion method of mechanical shearing was employed to treat tissue blocks. This approach is highly suitable for high-fibrosis tumors and tumors with tight intercellular connections, such as HNC. Since intense enzymatic digestion and dissociation processes were avoided, the ALI method better preserves the original tumor tissue structure and the diverse cell types, reducing the risk of clonal selection in the early stage of culture, and enabling the cultivated model to truly reflect the genetic and phenotypic heterogeneity of the primary tumor. In the ALI dual-disc system, the immune cells and the fibroblast matrix of tumor tissues could be maintained for a certain period of time, forming a complex ecological environment closer to the *in vivo* environment. This method provides a holistic strategy for *in vitro* immune TME modeling that can explore complex crosstalk among multiple cell populations, such as the impact of tumor-associated fibroblasts on tumor cell development.

Immunotherapy has been widely recognized as a new and effective approach for treating various cancers. Currently, ICIs (such as PD-1/PD-L1 inhibitors) are the main treatment for HNC, among which the anti-PD-1 ICI pembrolizumab is the first-line treatment. However, overall OOR of pembrolizumab as a single treatment is relatively low. Organoid models that can be used for preclinical research have been rapidly developed. They can be applied in screening chemotherapy and the usage of targeted drugs. However, most models cannot maintain the TME, so they cannot be used to screen immunotherapeutic drugs. HNC tumors are known to have significant individual differences, and T-cell-mediated tumor killing is influenced by multiple factors. Hence, the treatment of advanced HNC patients requires more personalized precision medicine. In this study, the ALI method was employed to study and establish organoids capable of maintaining the immune microenvironment and successfully preserving T lymphocytes, which is the most prominent advantage of the ALI culture method. We used pembrolizumab to treat organoids, and the organoids demonstrated different responses to immunotherapy. Twenty-eight organoid models responded to immunotherapy drugs (17.86%), which was similar to that reported in previous studies on OOR for HNCs, including squamous cell carcinoma and salivary gland carcinoma. The model has the potential to become an *in vitro* model for personalized precision medicine in tumor immunotherapy and combination therapy.

However, the ALI culture steps are more complicated than traditional methods. The ALI method requires the manual handling of tissue fragments and precise spreading on membranes; that is, the operators need higher technical skills. Thus, the technique is not easy to standardize. Traditional organoids can be cultured in 96- or 384-well plates, which are highly suitable for large-scale drug screening. ALI cultures usually employ insertable petri dishes, and each well needs to be treated separately, which limits throughput and increases cost. To address this challenge, future efforts should pursue innovation in the miniaturization of ALI-based tumor organoid platforms. This includes developing specialized microfluidic chips or miniaturized culture plates (e.g., hanging drop arrays) to enhance both throughput and compatibility. In addition, the typical expansion mode of ALI organoids is mainly the outward growth and proliferation of the original tissue blocks, rather than forming individual, passageable and expandable organoid spheres. Therefore, the amplification speed is relatively slow, making it difficult to obtain a large number of cells in a short period of time, which is challenging for experiments that require many cells, such as genomic sequencing. The tissue block grows on a porous membrane, and its 3D structure is less regular than organoid spheres in the matrix gel scaffold method, which poses certain difficulties for real-time imaging, size measurement, and automated analysis.

In this study, the application of ALI-HNCOs in cancer immunotherapy *in vitro* demonstrated that patient-derived HNC ALI-PDOs could simulate PD-1/PD-L1 checkpoint blockade. Moreover, treatment with the anti-PD-1 antibody pembrolizumab activated and proliferated TILs in the ALI-PDO model and triggered cytotoxic reactions, indicating its great potential and prospects for immunotherapeutic methods in the field of precision medicine. Our subsequent research will focus on and be devoted to the clinical validation and clinical translation of ALI culture models, and we also confirmed its utility as a screening platform for both chemotherapy and radiotherapy, comparable to traditional organoids, which facilitates the selection of optimized combination therapies. Since the cell preparation does not meet the level required by Good Clinical Practice (GCP) for clinical drug trials, experimental validation is needed in future research.

## Data Availability

The original contributions presented in the study are included in the article/supplementary material. Further inquiries can be directed to the corresponding author/s.
